# Impact of Menstrual Function on Hormonal Response to Repeated Bouts of Intense Exercise

**DOI:** 10.3389/fphys.2019.00942

**Published:** 2019-07-31

**Authors:** Anna K. Melin, Christian Ritz, Jens Faber, Sven Skouby, Jessica Pingel, Jorunn Sundgot-Borgen, Anders Sjödin, Åsa B. Tornberg

**Affiliations:** ^1^Department of Sport Science, Linnaeus University, Kalmar, Sweden; ^2^Department of Nutrition, Exercise and Sports, University of Copenhagen, Frederiksberg, Denmark; ^3^Faculty of Health and Medical Sciences, University of Copenhagen, Copenhagen, Denmark; ^4^Department of Endocrinology, Herlev Hospital, Herlev, Denmark; ^5^Endocrinological and Reproductive Unit, Department of Obstetrics and Gynecology, Herlev Hospital, Herlev, Denmark; ^6^Department of Neuroscience, University of Copenhagen, Copenhagen, Denmark; ^7^Norwegian School of Sport Sciences, Oslo, Norway; ^8^Department of Health Sciences, Lund University, Lund, Sweden

**Keywords:** amenorrhea, energy availability, overtraining syndrome, female athlete, brain derived neuronal factor

## Abstract

**Background:**

Strenous exercise stimulates the hypothalamic-pituitary (HP) axis in order to ensure homeostasis and promote anabolism. Furthermore, exercise stimulates a transient increase in the neurotrophin brain-derived neurotrophic factor (BDNF) suggested to mediate the anxiolytic effects of exercise. Athletes with secondary functional hypothalamic amenorrhea (FHA) have been reported to have lower BDNF, and a blunted HP axis response to exercise as athletes with overtraining syndrome.

**Aim:**

The aim of the study was to investigate the hormonal and BDNF responses to a two-bout maximal exercise protocol with four hours of recovery in between in FHA and eumenorrheic (EUM) athletes.

**Methods:**

Eumenorrheic (*n* = 16) and FHA (*n* = 14) endurance athletes were recruited from national teams and competitive clubs. Protocols included gynecological examination; body composition (DXA); 7-day assessment of energy availability; blood sampling pre and post the two exercises tests.

**Results:**

There were no differences between groups in hormonal responses to the first exercise bout. After the second exercise bout IGFBP-3 increased more in FHA compared with EUM athletes (2.1 ± 0.5 vs. 0.6 ± 0.6 μg/L, *p* = 0.048). There were non-significant trends toward higher increase in IGF-1 (39.3 ± 4.3 vs. 28.0 ± 4.6 μg/L, *p* = 0.074), BDNF (96.5 ± 22.9 vs. 34.4 ± 23.5 μg/L, *p* = 0.058), GH to cortisol ratio (0.329 ± 0.010 vs. 0.058 ± 0.010, *p* = 0.082), and decrease in IGF-1 to IGFBP-3 ratio (−2.04 ± 1.2 vs. 0.92 ± 1.22, *p* = 0.081) in athletes with FHA compared with EUM athletes. Furthermore, there was a non-significant trend toward a higher increase in prolactin to cortisol ratio in EUM athletes compared with athletes with FHA (0.60 ± 0.15 vs. 0.23 ± 0.15, *p* = 0.071). No differences in the hormonal or BDNF responses between the two exercise bouts as a result of menstrual function were found.

**Conclusion:**

No major differences in the hormonal or BDNF responses between the two exercise bouts as a result of menstrual function could be detected.

## Introduction

Exercise stimulates the hypothalamic-pituitary (HP) axis leading to increased levels of prolactin (Rojas et al., 2012), adrenocorticotropic hormone (ACTH), cortisol, growth hormone (GH), insulin-like growth hormone 1 (IGF-1), and insulin-like growth factor binding protein (IGFBP) in order to ensure homeostasis and promote anabolism (Mastorakos et al., 2005). However, a chronic mild hypercortisolism as well as a decreased HP-adrenal response to exercise has been reported in highly trained athletes (Mastorakos et al., 2005). A transient increase in brain-derived neurotrophic factor (BDNF) seems to be triggered by high intensity exercise. Neurotrophins including BDNF are involved in central and peripher cellular energy processes, such as enhanced skeletal muscle lipid oxidation, and benefits to mood and various cognitive domains as well as the growth, development, maintenance and function of several neuronal systems (Rojas et al., 2006; Knaepen et al., 2010; Skriver et al., 2014; Dinoff et al., 2017).

Low energy availability (LEA) is a common phenomenon in female athletes (Gibbs et al., 2013), and is the aetiological factor of Relative Energy deficiency in Sports (RED-S) with multiple adverse health outcomes. These include, but are not limited to, reproductive dysfunction, impaired bone health, immunity and protein synthesis, as well as elevations in cardiovascular risk factors and potential negative effects on performance (Mountjoy et al., 2018). LEA can be the result of eating disorders, intentional restriction in food in order to loose body weight, a misguided low energy dens diet, or unintentionally due to the lack of appetite in periods with high training volumes (Burke et al., 2018). In healthy women, 5 days of clinical LEA ( <125 kJ/kg FFM/day) (Gibbs et al., 2013) have been shown to alter the HP axis such as a reduction in the pulsatility of gonadotropin releasing hormone, and luteinizing hormone, with increased risk of functional hypothalamic amenorrhea (FHA) (Loucks and Thuma, 2003). Although both clinical and subclinical LEA ( <188 kJ/kg FFM/day) has been reported in female athletes with FHA (Gibbs et al., 2013; Melin et al., 2015), multiple alterations in HP axis with lowered fasting IGF-1 and prolactin levels, and increased cortisol levels have been observed, indicating an increased catabolic drive compared with eumenorrheic (EUM) athletes (Laughlin et al., 1998; Gordon, 2010; Warren, 2011; Melin et al., 2014; Tornberg et al., 2017).

In studies regarding different exercise intensities between FHA and EUM athletes, FHA athletes seem to have a blunted catecholamine (Schaal et al., 2011), and prolactin response (Loucks and Horvath, 1984; De Souza et al., 1991) following high-intensity exercise while both suppressed (Loucks and Horvath, 1984; De Souza et al., 1991), and increased cortisol response have been reported (Sanders et al., 2018). LEA and other stressors are proposed to inhibit proper recovery especially in periods with large training volumes, with increased risk of developing overtraining syndrome with long-term symptoms of fatigue, and decreased physical and cognitive performance (Meeusen et al., 2010; Cadegiani and Kater, 2017). Overtraining syndrome has been proposed to be identified by a blunted HP-axis hormonal response to a two-bout maximal exercise protocol (Meeusen et al., 2007; Nederhof et al., 2008). Accordingly, the aim of the present explorative study was to investigate potential differences in endocrine and BDNF responses to a two-bout maximal exercise protocol between FHA and EUM endurance athletes. Our hypothesis was that the athletes with FHA would have a more blunted endocrine and BDNF response to the second exercise test compared with the eumenorrheic athletes.

## Materials and Methods

Most of the protocols and methods used in this study have previously been described in detail (Melin et al., 2014, 2015; Tornberg et al., 2017) but, briefly, competitive athletes (18–38 years) were recruited through the Danish and Swedish endurance sport federations (middle and long distance running, orienteering and triathlon), and through local competitive endurance sports clubs in the Oresund region of Denmark and Sweden. Permission to undertake the study was provided by the Data Inspectorate and the Regional Ethical Committees in both Sweden and Denmark (Nos. 2011/576 and H-4-2011-096) and endorsed by the Swedish and Danish Confederations of Sports and Team Denmark. The protocol was registered at www.clinicaltrials.gov. All testing complied with the 1964 Declaration of Helsinki.

Exclusion criteria included pregnancy, chronic illness, smoking, use of hormonal contraceptives including local intrauterine hormone releasing systems 6 weeks prior to investigation, injuries preventing the athlete from exercise training ≥2 weeks, or menstrual dysfunctions other than secondary FHA. Menstruating athletes were examined and all tests performed in the early follicular phase, on the second to the fifth day of bleeding to eliminate hormonal fluctuations during the luteal phase, and thereby limiting the variability between subjects. All tests were performed on two consecutive days. The 1st day included the gynecological *trans* -vaginal ultrasound examination (Ultrasound Scanner, Class 1 type B, B-K Medical Ref Type 2202), menstrual anamnesis performed by a skilled gynecologist, and blood sex hormones, as well as body composition assessment by a trained technician by Dual-energyX-ray absorptiometry (DXA) (Hologic Model Discovery A 2009, QDR series Hologic Inc., Waltham, MA, United States) in the fasted, rested state, according to standard operation procedures. The 2nd day included assessment of aerobic capacity and hormonal response to incremental exercise tests. Energy intake was assessed by a prospective weighed food record for 7 consecutive days in the athletes home environment, simultaneously with exercise energy expenditure assessments by heart rate monitors and detailed training logs including type, duration, and intensity of exercise. Subjects were instructed to maintain their normal food habits, eating patterns and training regime during this period. Energy expenditure from the 7 days of training was averaged to determine exercise energy expenditure for each subject, and used to calculate EA. Individual prediction equations from measured heart rate and corresponding energy expenditure during the incremental maximal exercise test in the laboratory provided the basis for the calculation of exercise energy expenditure, using the training log and heart rate measurement for each training session. Regression lines were calculated for corresponding values of heart rate and energy expenditure during the exercise test in the laboratory, and for the recorded heart rate during all exercise sessions. EA was calculated by subtracting mean exercise energy expenditure from the mean energy intake then calculated relative to FFM. In order not to overestimate exercise energy expenditure and thereby underestimate EA, exercise energy expenditure was corrected for the mean total energy expenditure (measured resting metabolic rate by ventilated hood and non-exercise activity thermogenesis by accelerometer) without exercise energy expenditure during the equivalent time period. Athletes retrospectively classified as having EUM (*n* = 16) (menstrual cycles of 28 ± 7 days and sex hormones within the normal range) and FHA (*n* = 14) (absence of at least three consecutive menstrual cycles where other causes than hypothalamic suppression had been ruled out) (Nattiv et al., 2007) were included in the analysis. Detailed information regarding gynecological assessment and characterization is to be found in Melin et al. (2015), Tornberg et al. (2017). Baseline characteristics regarding the participants age, anthropometrics, EA, reproductive function, training amounts and oxygen consumption have previously been published (Melin et al., 2014, 2015; Tornberg et al., 2017).

### The Two-Bout Exercise Protocols

#### Aerobic Performance and Hormonal Response

Two incremental exercise tests (T^1^ and T^2^) on a cycle ergometer were performed with four hours of recovery in between according the protocol by Meeusen et al. (2010), since this protocol has been reported to provide a good indication of the normal stress related HP-axis response in athletes, with a suppressed HP-axis response to the second exercise bout in athletes with over training syndrome. The tests were performed at 11.00 a.m. and 3.00 p.m, two hours after a standardized breakfast and lunch containing no more than 650 kcal, respectively. The tests were initiated by cycling for 6 min at 50 W, followed by an increase in workload of 12–14 W per minute until exhaustion, with a cadence of 60 rpm. An airtight mask covering the mouth and nose was worn in order to measure breath-by-breath VO_2_ and VCO_2_, for determination of VO_2peak_ (Oxycon Pro 4, Jaeger, Germany).

#### Blood Analysis

Venus blood was drawn before and as fast as possible after the exercises test with the athlete still sitting on the cycle ergometer. All blood samples were first stored at 4°C and then centrifuged and seperated. Serum and plasma were allocated into separate tubes to be stored at −80°C. All analyses were performed at the same time in the same badge.

Pre and post both incremental exercise bouts prolactin was analyzed using ADVIA Centaur Immunoassay (Systems Siemens Healthcare Diagnostics Products GmbH, Germany) with analytic sensitivity of 6.4 to 4240 μ IU/mL and intra- inter-assay coefficient of variation (CV) of 2.0 and 2.3%, respectively. ACTH levels were detected using a two-site ELISA [(ABNOVA, ACTH human ELISA kit, CAT. No. KA0916) Abnova Taiwan Cooperation, Taipei, Taiwan] with analytic sensitivity of 1.5 to 1045 pg/mL and intra-inter assay CV of 3.7 and 6.0%. Cortisol, GH, IGF-1, and IGFBP-3 were analyzed using Roche Electro Chemiluminescence Immunoassay (ECLI; Roche Diagnostic, Bromma, Sweden) with an analytical sensitivity for the cortisol assay of 0.5 to 1750 nmol/L, for the GH and IGF-1 assay of 0.03 to 50 μg/L, and for the IGFBP-3 assay of 0.08 to 10 μg/L, with an intra- and interassay CV of 1.3 and 2.1% (cortisol), 3% (GH), 2.0 and 2.5% (IGF-1), and 8% (IGFBP-3). Insulin was analyzed using Access Ultrasensitive Insulin assay (Beckman Coulter, Bromma, Sweden) with an analytic sensitivity of 0.03–50 μg/L and an assay CV of 7 and 8%, respectively. BDNF levels were detected using a sandwich enzyme-linked immunosorbent assay [Chemikine^TM^ BDNF sandwich ELISA (CAT. No. CYT306)], Millipore A/S, Hellerup DK, with an analytic sensitivity of 7.8 to 500 pg/mL and intra-inter assay CV of 3.7 and 8.5 %. All analysis was conducted in accordance to the manufacturer’s protocol.

### Statistics

Participants were divided into two groups by their reproductive function: EUM or FHA. Baseline characteristics were summarized using mean ± SD. Hormonal responses were described by means of linear mixed models with the three-way interaction between time-test-reproductive function group as fixed effect and participants as random effects. Changes from before to post exercise tests within and between groups were estimated and compared in *post hoc* *t*-tests based on the model fits. No adjustment for multiplicity was applied. Statistical significance was defined as *p* < 0.05. All statistical analyses were performed using R (R Core Team, 2018).

## Results

Athletes with FHA had a lower total body mass and fat mass compared to EUM (Table 1), but no differences in aerobic capacity or in total or aerobic weekly training time between the groups were found. Furthermore, athletes with FHA had lower FFM compared with EUM athletes, despite the latter group performing less resistance training. Nineteen participants had subclinical LEA (ten FHA and eight EUM), during the week of assessment of which six had clinical LEA (three FHA and three EUM athletes).

**TABLE 1:** Baseline characteristics of participants according to reproductive function.

	**EUM (*n* = 16)**	**FHA (*n* = 14)**	p-value
Age (years)	27.6 ± 5.6	26.1 ± 5.6	0.46
Height (cm)	169 ± 5	167 ± 6	0.38
Body mass (kg)	60.2 ± 7.1	55.0 ± 5.8	0.04
BMI (kg/m^2^)	21.0 ± 2.0	19.6 ± 1.8	0.05
Fat mass (kg)	12.8 ± 3.2	10.1 ± 2.6	0.02
Fat mass (%)	20.9 ± 3.5	18.3 ± 3.1	0.04
FFM (kg)	47.8 ± 4.8	44.9 ± 4.0	0.08
FFM (%)	79.1 ± 3.5	81.8 ± 3.2	0.04
Energy availability (kJ/kg FFM)	163 ± 13	171 ± 15	0.68
VO_2peak_ (L/min)	3.19 ± 0.38	3.13 ± 0.44	0.67
Exercise (min/week)	661 ± 240	721 ± 300	0.59
Aerobic exercise (min/week)	566 ± 312	429 ± 326	0.25
Resistance training (min/week)	49 ± 54	128 ± 112	0.02

*Data shown as mean ± SD. EUM, eumenorrheic athletes; FHA, functional hypothalamic amenorrhoeic athletes; BMI, body mass index; FFM, fat free mass.*

### Hormonal Levels at Baseline

At baseline only prolactin levels differed between groups, being lower in athletes with FHA (Table 2).

**TABLE 2:** Baseline hormonal levels according to reproductive function.

	**EUM (*n* = 16)**	**FHA (*n* = 14)**	p-value
ACTH (μg/L)	18.7 ± 9.9	21.9 ± 7.8	0.35
Cortisol (nmol/L)	342 ± 119	375 ± 69	0.38
GH (μg/L)	0.71 ± 0.32	2.2 ± 3.7	0.15
IGF-1 (μg/L)	231 ± 51	219 ± 58	0.55
IGFBP-3 (μg/L)	5.4 ± 0.6	5.4 ± 1.6	0.95
Insulin (pmol/L)	107 ± 38	118 ± 68	0.60
Glucose (nmol/L)	4.8 ± 0.7	4.3 ± 0.9	0.60
Prolactin (μg/L)	231 ± 90	145 ± 28	<0.01
BDNF (μg/L)	113.7 ± 90.1	82.5 ± 64.3	0.30

*Data shown as mean ± SD. ACTH, adrenocorticotropic hormone; BDNF, brain-derived neurotrophic factor; cortisol, GH, growth hormone; IGF-1, insulin-like growth factor 1; IGFBP3, insulin-like growth factor-binding protein 3 in athletes with EUM, eumenorrhea; FHA, functional hypothalamic amenorrhea.*

### Endocrine Response to the Incremental Exercise Tests

There were no differences in the hormonal response to the first exercise bout between groups (Table 3). However, following the second bout, IGFBP-3 increased more in FHA compared with EUM athletes, with a similar trend for IGF-1 and BDNF (Table 4). There was also a trend toward a higher increase in GH to cortisol ratio, and decrease in IGF-1 to IGFBP-3 ratio in FHA athletes compared with EUM athletes. There was furthermore a trend toward a higher increase in prolactin to cortisol ratio in EUM compared with FHA athletes. There were no differences in the hormonal responses between Test^1^ and Test^2^ as a result of menstrual function (Table 5).

**TABLE 3:** Comparison of changes in hormonal responses from pre to post Test^1^, within and between reproductive function groups.

	**Test^1^: change from pre to post within and between groups**					
	**Within groups**				**Between groups**	
	**EUM (*n* = 16)**		**FHA (*n* = 14)**			
		p-Value		p-Value		p-Value
Prolactin (μg/L)	68.6 ± 34.1	0.044	108.2 ± 35.7	0.002	−39.7 ± 49.1	0.419
ACTH (μg/L)	65.9 ± 12.4	<0.001	68.7 ± 12.5	<0.001	2.8 ± 17.6	0.874
Cortisol (nmol/L)	7.6 ± 37.5	0.838	79.5 ± 38.9	0.041	−71.8 ± 53.9	0.183
GH (μg/L)	3.7 ± 2.1	0.077	4.1 ± 2.1	0.049	−0.4 ± 2.9	0.881
IGF-1 (μg/L)	32.8 ± 4.3	<0.001	36.5 ± 4.3	<0.001	−3.7 ± 6.1	0.545
IGFBP-3 (μg/L)	0.9 ± 0.5	0.090	0.7 ± 0.5	0.218	0.2 ± 0.8	0.743
Insulin (pmol/L)	−58.3 ± 14.2	<0.001	−62.5 ± 14.2	<0.001	4.2 ± 20.0	0.833
Prolactin:cortisol	0.27 ± 0.15	0.061	0.16 ± 0.152	0.278	01.1 ± 0.21	0.605
Cortisol:insulin	0.007 ± 0.003	0.011	0.009 ± 0.003	0.001	−0.002 ± 0.004	0.605
GH:cortisol	0.010 ± 0.010	0.338	0.003 ± 0.01	0.757	0.006 ± 0.014	0.659
GH:insulin	0.852 ± 1.39	0.540	0.985 ± 1.39	0.479	−0.133 ± 1.966	0.946
IGF-1:IGFBP-3	−0.88 ± 1.17	0.452	0.11 ± 1.17	0.922	−0.99 ± 1.65	0.548
IGF:cortisol	0.187 ± 0.093	0.045	0.014 ± 0.097	0.884	0.173 ± 0.134	0.198
BDNF (μg/L)	3.6 ± 22.9	0.875	41.9 ± 23.5	0.074	−38.3 ± 32.8	0.243

*Data shown as mean and standard error. EUM, eumenorrheic athletes; athletes with FHA, functional hypothalamic amenorrhea; ACTH, adrenocorticotropic hormone; BDNF, brain derived neurotrophic factor; GH, growth hormone; IGF-1, insulin like growth factor 1; IGFBP-3, insulin like growth factor binding protein 3.*

**TABLE 4:** Comparison of changes in hormonal responses from pre to post Test^2^, within and between reproductive function groups.

	**Test^2^: change from pre to post within and between groups**					
	**Within group**				**Between groups**	
	**EUM (*n* = 16)**		**FHA (*n* = 14)**			
		p-Value		p-Value		p-Value
Prolactin (μg/L)	176.9 ± 34.1	<0.001	148.4 ± 34.1	<0.001	28.5 ± 48.2	0.554
ACTH (μg/L)	67.2 ± 12.1	<0.001	65.1 ± 12.5	<0.001	−2.1 ± 17.4	0.902
Cortisol (nmol/L)	53.8 ± 37.5	0.151	98.6 ± 37.5	0.008	−44.9 ± 53.0	0.397
GH (μg/L)	15.7 ± 2.2	<0.001	12.9 ± 2.1	<0.001	2.8 ± 3.0	0.355
IGF-1 (μg/L)	28.0 ± 4.6	<0.001	39.3 ± 4.3	<0.001	−11.3 ± 6.3	0.074
IGFBP-3 (μg/L)	0.6 ± 0.6	0.321	2.1 ± 0.5	<0.001	−1.5 ± 0.8	0.048
Insulin (pmol/L)	6.2 ± 14.8	0.675	13.7 ± 14.2	0.332	−7.5 ± 20.5	0.712
Prolactin:cortisol	0.60 ± 0.15	<0.001	0.23 ± 0.15	0.116	0.37 ± 0.21	0.071
Cortisol:insulin	0.004 ± 0.003	0.202	0.008 ± 0.003	0.005	−0.004 ± 0.004	0.310
GH:cortisol	0.058 ± 0.010	<0.001	0.329 ± 0.010	0.001	0.025 ± 0.014	0.082
GH:insulin	5.318 ± 1.450	<0.001	4.376 ± 1.391	0.002	0.942 ± 2.008	0.639
IGF-1:IGFBP-3	0.92 ± 1.22	0.454	−2.04 ± 1.2	0.080	2.96 ± 1.70	0.081
IGF:cortisol	0.001 ± 0.098	0.991	−0.051 ± 0.093	0.585	0.052 ± 0.135	0.700
BDNF (μg/L)	34.4 ± 23.5	0.143	96.5 ± 22.9	<0.001	−62.1 ± 32.8	0.058

*Data shown as mean and standard error. EUM, Eumenorrheic athletes; athletes with FHA, functional hypothalamic amenorrhea; ACTH, adrenocorticotropic hormone; BDNF, brain derived neurotrophic factor; GH, growth hormone; IGF-1, insulin like growth factor 1; IGFBP-3, insulin like growth factor binding protein 3.*

**TABLE 5:** Comparison of hormonal responses between Test^1^ and Test^2^, within and between reproductive function groups.

	**Difference in change from pre to post between Test^1^ and Test^2^**					
	**Within groups**					
	**EUM (*n* = 16)**		**FHA (*n* = 14)**		**Between groups**	
		p-Value		p-Value		p-Value
Prolactin (μg/L)	108.4 ± 48.2	0.025	40.2 ± 49.1	0.413	68.2 ± 68.8	0.322
ACTH (μg/L)	1.33 ± 17.3	0.939	−3.61 ± 7.7	0.839	4.94 ± 24.8	0.842
Cortisol (nmol/L)	46.1 ± 55.0	0.384	19.2 ± 54.0	0.722	27.0 ± 75.6	0.721
GH (μg/L)	12.0 ± 3.0	0.003	8.8 ± 2.9	<0.001	3.2 ± 4.2	0.447
IGF-1 (μg/L)	−4.8 ± 6.3	0.451	2.8 ± 6.1	0.650	−7.5 ± 8.8	0.391
IGFBP-3 (μg/L)	−0.3 ± 0.8	0.652	1.4 ± 0.8	0.058	−1.8 ± 1.1	0.100
Insulin (pmol/L)	64.5 ± 20.5	0.002	76.2 ± 20.0	<0.001	−11.8 ± 28.6	0.681
Prolactin:cortisol	0.33 ± 0.21	0.111	0.06 ± 0.21	0.758	0.26 ± 0.29	0.370
Cortisol:insulin	−0.003 ± 0.004	0.401	−0.001 ± 0.004	0.734	−0.002 ± 0.006	0.723
GH:cortisol	0.048 ± 0.014	<0.001	0.030 ± 0.014	0.039	0.019 ± 0.204	0.358
GH:insulin	4.466 ± 0.262	0.026	3.391 ± 1.967	0.085	1.074 ± 2.811	0.702
IGF-1:IGFBP-3	1.80 ± 1.70	0.289	−2.16 ± 1.65	0.192	3.95 ± 2.37	0.095
IGF:cortisol	−0.186 ± 0.135	0.170	−0.065 ± 0.134	0.629	−0.121 ± 0.190	0.527
BDNF (μg/L)	30.8 ± 32.8	0.348	54.6 ± 32.8	0.096	−23.8 ± 46.4	0.608

*Data shown as mean and standard error. EUM, Eumenorrheic athletes; athletes with FHA, functional hypothalamic amenorrhea; ACTH, adrenocorticotropic hormone; BDNF, brain derived neurotrophic factor; GH, growth hormone; IGF-1, insulin like growth factor 1; IGFBP-3, insulin like growth factor binding protein 3.*

## Discussion

This explorative study investigated the differences in hormonal and BDNF responses to strenuous exercise in FHA and EUM endurance athletes. In this cohort of intensive training women with either FHA or EUM, current subclinical LEA was found in 93 and 75%, respectively. Athletes with FHA were characterized by lower total body mass, lower absolute fat mass and FFM. Athletes with FHA and EUM trained equally regarding aerobic exercise, but EUM performed less resistant training.

The main finding was that there were no major differences in the hormonal responses between the two exercise bouts as a result of menstrual function. Thus, no signs of overtraining syndrome in the form of blunted hormonal responses after a second bout of exercise were found in these women, regardless of menstrual status.

Catecholamines increases the cardiac output and blood flow to the muscles, and cortisol with its principal regulator ACTH is secreted by the adrenal cortex to increase blood glucose levels to support the nutrient requirements of the central nervous system during stress, such as prolonged exercise, starvation, and hepatic glycogen depletion (Schaal et al., 2011; Elnour, 2018). Long-time exposure to stress hormones is also associated with increased proteolysis (Darmaun et al., 1988). Elevated fasting cortisol levels have earlier been reported in this group of athletes with FHA compared with EUM athletes (Melin et al., 2015), potentially explaining the lower FFM in athletes with FHA despite more resistance training compared with EUM athletes. However, two hours after a standardized breakfast (at 11 a.m.) we found no differences in non-fasting cortisol levels between the groups, which is in line with earlier findings reporting similar cortisol levels between athletes with FHA and EUM (De Souza et al., 1991; Schaal et al., 2011). In the present study we found no differences in the exercise induced cortisol responses between FHA and EUM in contrast to studies reporting blunted catecholamine (norepinephrine and epinephrine) and cortisol responses to a sub maximal exercise test in athletes with FHA compared with EUM athletes (Loucks and Horvath, 1984; De Souza et al., 1991; Schaal et al., 2011).

Growth hormone is essential for muscle growth mediated by IGF-1, and lowered IGF-1 levels are strongly associated with energy deficiency and has been reported in male cyclists after an ultra-endurance event (Geesmann et al., 2017), and in adolescent male wrestlers during a competitive wrestling season (Roemmich and Sinning, 1997). Increased GH secretion has also been reported in female athletes compared with non-athletes (Laughlin and Yen, 1996), and in patients with anorexia nervosa with systemic low IGF-1 levels, indicating a hepatic GH resistance (Roemmich and Sinning, 1997; Misra and Klibanski, 2014). Although similar IGF-1 levels have been reported in FHA and EUM athletes and non-athletes, higher IGFBP levels, and thereby a lower IGF-1 to IGFBP ratio have been reported in FHA athletes which has been suggested to act as an energy-conserving strategy by minimizing the hypoglycemic action of IGF-I (Laughlin and Yen, 1996). In the present study we found no difference in the exercise induced response in either GH, IGF-1 levels or GH to cortisol and GH to insulin ration between groups. However, the tendency toward an increased IGF-1 to cortisol ration in EUM athletes compared with FHA athletes after the second exercise bout might indicate a more anabolic response in EUM athletes, while the increased IGFBP-3 levels in FHA athletes compared with EUM athletes might indicate a more catabolic response to strenuous exercise in FHA athletes. Therefore, more studies investigating potential differences in anabolic response to exercise between EUM and FHA athletes are needed.

Prolactin is a sex hormone involved in diverse physiological processes, including angiogenesis, immune response, osmoregulation, and brain functions such as cognition and memory as well as having neuroprotective functions (Cabrera-Reyes et al., 2017). In the present study, baseline prolactin levels were lower in FHA compared with EUM athletes, but no differences in the exercise induced response between FHA and EUM athletes were found which is in contrast to the findings of De Souza et al. who reported a blunted prolactin response to exercise in FHA compared with EUM athletes (Loucks and Horvath, 1984; De Souza et al., 1991). However, we did find a more pronounced exercise induced increase in the prolactin to cortisol ratio in EUM athletes compared with athletes with FHA, indicating a more profound anabolic response.

Brain-derived neurotrophic factor is an essential neurotrophin connected with central and peripheral molecular processes of energy metabolism and homeostasis (Knaepen et al., 2010). A transient increase in BDNF triggered by high intensity exercise has been reported (Knaepen et al., 2010), and is suggested to mediate the beneficial antidepressant and anxiolytic effects of exercise (Antunes et al., 2016), and is positively correlated with cognitive performance (Heijnen et al., 2015). Lower fasting concentration of BDNF has earlier been reported in women with FHA compared with EUM women assessed in the follicular phase between the 8th and 12th day of the cycle (Podfigurna-Stopa et al., 2013). The exercise induced BDNF increase is reported to be affected by various factors such as exercise regimen and hormonal changes during the menstrual cycle, with a decrease in BDNF release during the luteal phase and increase during the follicular phase (Heijnen et al., 2015). In the present study, however, no differences in baseline BDNF levels were found between FHA and EUM athletes assessed in the early follicular phase (2nd to 5th day of bleeding), and exercise induced increase was only seen in FHA athletes.

### Limitations

The strength of this study is the thorough gynecological characterization, and that standardized protocols were followed in all examinations and tests. However, the lack of continuous assessment of ovulation in eumenorrheic subjects is a limitation since subclinical menstrual dysfunction due to LEA cannot be ruled out, especially since current subclinical and clinical LEA was equally prevalent in eumenorrheic and FHA participants. A further limitation is the low number of participants, which means a potential lack of ability to detect differences among the many secondary outcomes for which no prior sample size calculation was carried out. Moreover, the high number of variables assessed and statistically tested in the present study also increases the risk of type 1 errors. Thus the reported findings should mostly be viewed as exploratory and hypothesis generating.

## Conclusion

No major differences in the hormonal or BDNF responses between the two exercise bouts as a result of menstrual function were found in this study.

## Data Availability

The datasets for this study will not be made publicly available because this was not included in the ethical approval at the time.

## Ethics Statement

This study was carried out in accordance with the Declaration of Helsinki with written informed consent from all subjects. The protocol was approved by the Regional Ethical Committees in both Sweden and Denmark (Nos. 2011/576 and H-4-2011-096). Permission to undertake the study was provided by the Data Inspectorate and the endorsed by the Swedish and Danish Confederations of Sports and Team Denmark. The protocol was registered at www.clinicaltrials.gov.

## Author Contributions

AM, ÅT, JS-B, SS, and AS planned the study. AM, ÅT, JF, and SS collected the results. CR analyzed the data. AM, ÅT, JF, and AS wrote the manuscript. All authors accepted the manuscript before its submission.

## Conflict of Interest Statement

The authors declare that the research was conducted in the absence of any commercial or financial relationships that could be construed as a potential conflict of interest.
